# The spine-brain axis: is spinal anatomy associated with brain volume?

**DOI:** 10.1093/braincomms/fcae365

**Published:** 2024-10-22

**Authors:** Sergio Grosu, Trayana Nikolova, Roberto Lorbeer, Veit M Stoecklein, Susanne Rospleszcz, Nicola Fink, Christopher L Schlett, Corinna Storz, Ebba Beller, Daniel Keeser, Margit Heier, Lena S Kiefer, Elke Maurer, Sven S Walter, Birgit B Ertl-Wagner, Jens Ricke, Fabian Bamberg, Annette Peters, Sophia Stoecklein

**Affiliations:** Department of Radiology, LMU University Hospital, LMU Munich, 81377 Munich, Germany; Department of Radiology, LMU University Hospital, LMU Munich, 81377 Munich, Germany; Department of Radiology, LMU University Hospital, LMU Munich, 81377 Munich, Germany; Department of Neurosurgery, LMU University Hospital, LMU Munich, 81377 Munich, Germany; German Research Center for Environmental Health, Institute of Epidemiology, Helmholtz Center Munich, 85764 Neuherberg, Germany; Department of Epidemiology, Biometry and Epidemiology, Institute for Medical Information Processing, LMU Munich, 81377 Munich, Germany; Department of Radiology, LMU University Hospital, LMU Munich, 81377 Munich, Germany; Department of Diagnostic and Interventional Radiology, Medical Center-University of Freiburg, Faculty of Medicine, University of Freiburg, 79106 Freiburg, Germany; Department of Neuroradiology, Medical Center-University of Freiburg, Faculty of Medicine, University of Freiburg, 79106 Freiburg, Germany; Paediatric Radiology and Neuroradiology, Institute of Diagnostic and Interventional Radiology, University Medical Centre Rostock, 18057 Rostock, Germany; Department of Radiology, LMU University Hospital, LMU Munich, 81377 Munich, Germany; Department of Psychiatry, LMU University Hospital, LMU Munich, 80336 Munich, Germany; German Research Center for Environmental Health, Institute of Epidemiology, Helmholtz Center Munich, 85764 Neuherberg, Germany; KORA Study Centre, University Hospital of Augsburg, 86153 Augsburg, Germany; Department of Diagnostic and Interventional Radiology, University of Tuebingen, 72076 Tuebingen, Germany; Department of Nuclear Medicine and Clinical Molecular Imaging, University of Tuebingen, 72076 Tuebingen, Germany; Department for Trauma and Reconstructive Surgery, BG Unfallklinik Tuebingen, University of Tuebingen, 72076 Tuebingen, Germany; KORA Study Centre, University Hospital of Augsburg, 86153 Augsburg, Germany; Department of Radiology, Division of Musculoskeletal Radiology, New York University, Grossman School of Medicine, New York, NY 10016, USA; Department of Radiology, LMU University Hospital, LMU Munich, 81377 Munich, Germany; Department of Diagnostic Imaging, The Hospital for Sick Children, University of Toronto, Toronto, ON, Canada, M5G 1E8; Department of Medical Imaging, University of Toronto, Toronto, ON, Canada, M5T 1W7; Department of Radiology, LMU University Hospital, LMU Munich, 81377 Munich, Germany; Department of Diagnostic and Interventional Radiology, Medical Center-University of Freiburg, Faculty of Medicine, University of Freiburg, 79106 Freiburg, Germany; German Research Center for Environmental Health, Institute of Epidemiology, Helmholtz Center Munich, 85764 Neuherberg, Germany; Department of Epidemiology, Biometry and Epidemiology, Institute for Medical Information Processing, LMU Munich, 81377 Munich, Germany; German Center for Diabetes Research (DZD), 85764 Neuherberg, Germany; Department of Radiology, LMU University Hospital, LMU Munich, 81377 Munich, Germany

**Keywords:** spine, spinal canal, scoliosis, brain, magnetic resonance imaging

## Abstract

First small sample studies indicate that disturbances of spinal morphology may impair craniospinal flow of cerebrospinal fluid and result in neurodegeneration. The aim of this study was to evaluate the association of cervical spinal canal width and scoliosis with grey matter, white matter, ventricular and white matter hyperintensity volumes of the brain in a large study sample. Four hundred participants underwent whole-body 3 T magnetic resonance imaging. Grey matter, white matter and ventricular volumes were quantified using a warp-based automated brain volumetric approach. Spinal canal diameters were measured manually at the cervical vertebrae 2/3 level. Scoliosis was evaluated using manual measurements of the Cobb angle. Linear binomial regression analyses of measures of brain volumes and spine anatomy were performed while adjusting for age, sex, hypertension, cholesterol levels, body mass index, smoking and alcohol consumption. Three hundred eighty-three participants were included [57% male; age: 56.3 (±9.2) years]. After adjustment, smaller spinal canal width at the cervical vertebrae 2/3 level was associated with lower grey matter (*P* = 0.034), lower white matter (*P* = 0.012) and higher ventricular (*P* = 0.006, inverse association) volume. Participants with scoliosis had lower grey matter (*P* = 0.005), lower white matter (*P* = 0.011) and larger brain ventricular (*P* = 0.003) volumes than participants without scoliosis. However, these associations were attenuated after adjustment. Spinal canal width at the cervical vertebrae 2/3 level and scoliosis were not associated with white matter hyperintensity volume before and after adjustment (*P* > 0.864). In our study, cohort smaller spinal canal width at the cervical vertebrae 2/3 level and scoliosis were associated with lower grey and white matter volumes and larger ventricle size. These characteristics of the spine might constitute independent risk factors for neurodegeneration.

## Teaser

Grosu *et al*. evaluated the association of spinal anatomy with brain volumes. They found that smaller spinal canal width at the C2/3 level and scoliosis were associated with lower grey and white matter volumes and larger ventricle size. These characteristics of the spine might constitute risk factors for neurodegeneration.

## Graphical Abstract

**Graphical Abstract:**
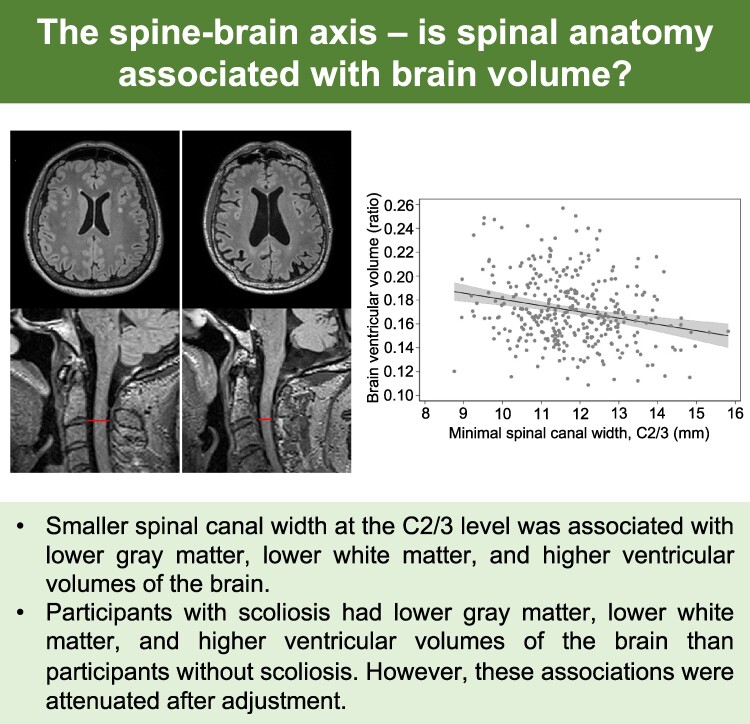

## Introduction

The CSF system closely interconnects the spine and brain.^1,2^ CSF protects the brain in different ways, providing supply of nutrients, metabolic homoeostasis, regulation of intracranial pressure and serving an important function in the removal of waste products.^1-3^ The spinal CSF space serves as a buffer system to compensate for increased intracranial pressure during systole, when arterial inflow causes a short-term volume challenge, and continues during diastole, when venous outflow leaves space for the CSF to flow through the spinal subpial space, thanks to an interplay between the arterial expansion and volume changes of the CSF spaces and veins.^4-6^

The importance of CSF dynamics for brain health is currently receiving attention as recent studies demonstrated that physiologic CSF circulation is responsible for brain regeneration.^7,8^ Unrestricted CSF flow between the cranial and spinal CSF compartments, along with other drainage mechanism such as the glymphatic system, Pacchioni granulations and transependymal resorption, could be an important component in maintaining brain health.^9-12^

Morphological features of the spine such as spinal canal diameter and scoliosis may impair systolic CSF outflow into the spinal canal. In animal models, cervical spinal canal stenosis has been associated with hydrocephalus development.^13,14^ A pilot study of 42 participants showed that maximal systolic craniospinal CSF flow rates were significantly reduced and spinal canal diameters at the cervical vertebrae (C) 2/3 level significantly smaller in patients with idiopathic normal pressure hydrocephalus. A significantly positive correlation between maximal CSF flow rates and spinal canal width at the C2/3 level was found.^15^ Furthermore, reduced spinal canal width could potentially impair the buffer function of CSF during systole and thus promote the development of white matter hyperintensities (WMHs) through increased intracranial blood pressure.^16^ However, despite potential implications for neurodegeneration, data on the relationship between brain volumes and spinal canal anatomy are scarce, especially assessing larger study samples.

We therefore aimed to evaluate potential associations between spinal canal diameter at the C2/3 level and scoliosis with grey matter, white matter, ventricular and WMH volumes of the brain in a large sample from the general population in the region of Augsburg, Germany.

## Materials and methods

### Study design and participants

This study was performed according to the Declaration of Helsinki and approved by the ethics committee of the Bavarian Chamber of Physicians, Munich (S4: EC No. 99186 and for genetic epidemiological questions 05004, F4 and FF4: EC No. 06068). The MRI examination protocol was approved by the ethics committee of the Ludwig-Maximilian-University Hospital, Munich.

This study is a retrospective analysis of a prospective sample of 400 participants from the population-based KORA FF4 study (2013–14, 2279 participants) younger than 75 years and without myocardial infarction, stroke, peripheral artery disease, Type 1 diabetes, missing oral glucose tolerance test, poor overall health condition, contraindication to MRI or unwillingness to undergo MRI, as described in detail previously.^17,18^ In a prior study on this study population, we investigated WMH volumes of the brain in patients with pre-diabetes, diabetes and normoglycaemia,^19^ whereas in this manuscript, we evaluate the association of spinal canal width and scoliosis with grey matter, white matter, ventricular and WMH volumes of the brain.

### Health assessment

Covariates including measures of body weight, blood pressure, cholesterol levels, alcohol consumption and smoking were collected in a standardized manner within the KORA study design according to Holle *et al*.^17^

### MRI

Image acquisition was performed on a 3 T MRI scanner (Magnetom Skyra; Siemens Healthineers, Erlangen, Germany) with a whole-body coil-matrix system accordingly to Bamberg *et al*.^18^ All participants were positioned in supine position in the centre of the table with arms parallel to the body and parallel, slightly bent legs.^20^

### Volumetric assessment of grey matter, white matter and ventricles

Volumetric grey matter, white matter and ventricle measurements were obtained using a warp-based automated brain volumetric approach based on T2-weighted FLAIR images accordingly to Beller *et al*.^21^ To adjust for differences in head size, ratio-corrected brain volumes were calculated by dividing grey matter, white matter and ventricular volume by total intracranial volume.^21,22^ WMH volume could potentially influence white matter volume.

### WMH volume

Cerebral WMHs were manually segmented on T2-weighted FLAIR images reconstructed in axial plane accordingly to Grosu *et al*.^23^ Image analyses were performed blinded to all other measurements and all clinical data.

### Spinal canal diameter

Spinal canal diameters were manually measured in the midsagittal plane at the cervical vertebrae (C) 2/3 level from the ventral margin of the spinal canal (CSF column) at the height of the midpoint of the intervertebral disc space and drawn perpendicular to the anterior cord surface on T2-weighted FLAIR images.^24^ Additionally, spinal canal diameters of the lumbar vertebra (L) 1 to sacral vertebra (S) 1 levels were measured on T1-weighted VIBE DIXON images. Measurements were performed by a radiologist (S.G., 5 years of experience in neuroimaging) and edited where necessary by an independent radiologist (S.S., 8 years of experience in neuroimaging). To assess inter- and intra-reader variability, an independent radiologist (N.F., 5 years of experience in neuroimaging) evaluated a subsample of 60 participants. Inter- and intra-reader agreement for spinal canal diameter measurements was performed using the intra-class correlation coefficient (ICC). Image analyses were performed blinded to all other measurements and all clinical data. Repeated measurements for inter- and intra-reader assessment were performed blinded to the previous measurements, other measurements and all clinical data.

### Scoliosis

To evaluate scoliosis, the Cobb angle was assessed on T1-weighted VIBE DIXON images by a radiologist (S.S.W., 5 years of experience in musculoskeletal imaging) and independent trauma surgeon (E.M., 6 years of experience in musculoskeletal imaging) measuring the angle created by an extension line from the upper end-plate of the uppermost vertebral body and lower end-plate of the lowermost vertebral body involved in lateral spinal deviation (Supplementary Fig. 1).^25,26^ Scoliosis was defined as lateral spinal deviation with a Cobb angle ≥ 10°.^27^ To assess inter- and intra-reader variability, an independent radiologist (N.F., 5 years of experience in musculoskeletal imaging) evaluated a subsample of 60 participants. Inter- and intra-reader agreement for scoliosis measurements was performed using ICC. Image analyses were performed blinded to all other measurements and all clinical data. Repeated measurements for inter- and intra-reader assessment were performed blinded to the previous measurements, other measurements and all clinical data.

### Statistical analysis

Characteristics of the study population were summarized by arithmetic means with standard deviation for continuous variables or counts and percentages for categorical variables.

**Figure 1:**
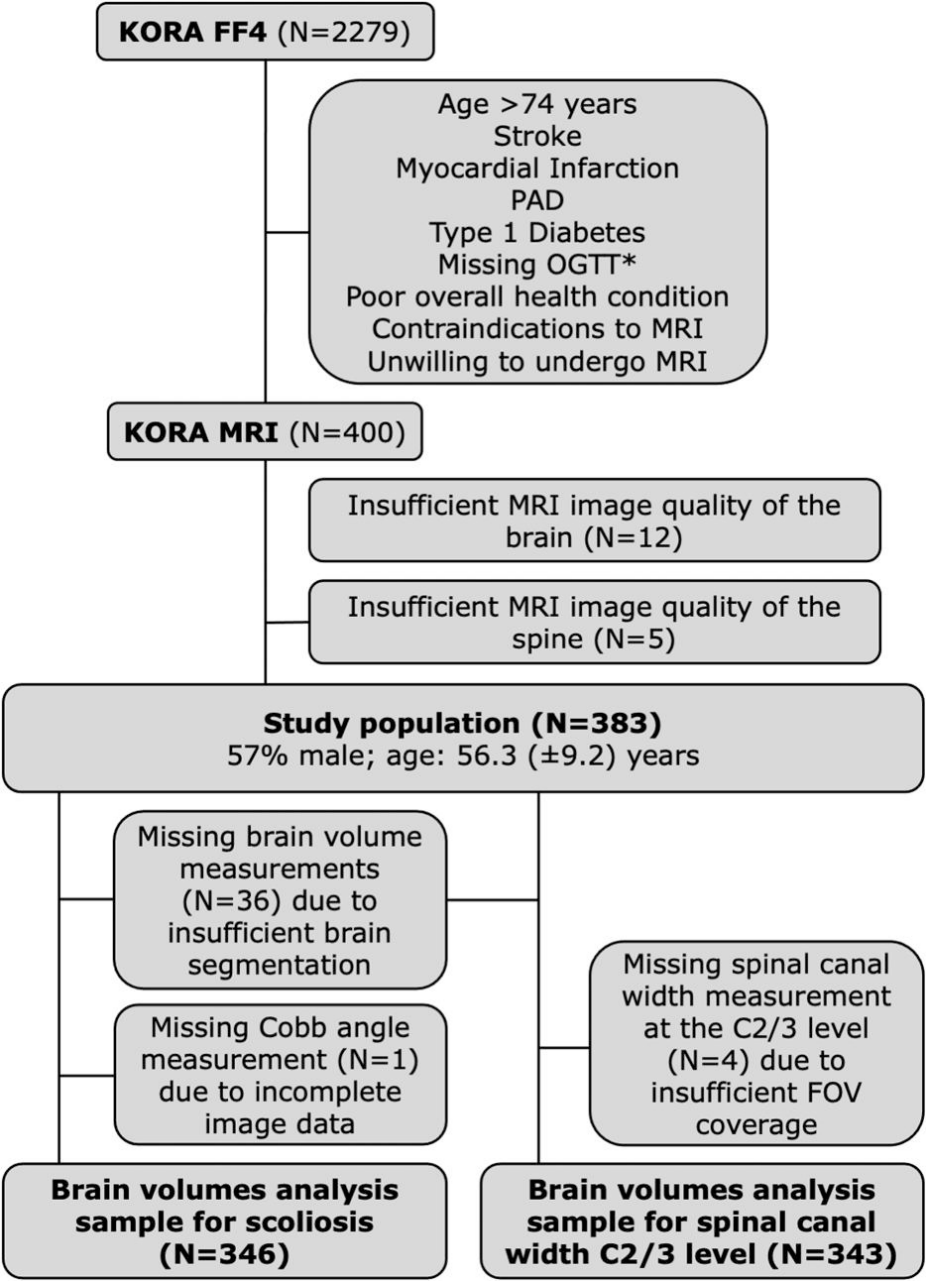
**Flow diagram of the study population.** C, cervical vertebrae; FOV, field of view; OGTT, oral glucose tolerance test; PAD, peripheral artery disease.

Normal distribution of variables was tested graphically and by Shapiro–Francia W test. Unadjusted comparisons of brain volume parameters between participants without and with scoliosis were displayed as boxplots and tested by *t*-test. The associations between spinal canal diameter as well as scoliosis (independent variables) and total grey matter volume, total white matter volume and brain ventricular volume (dependent variables) were evaluated by separated univariate linear regression analysis with estimated β-coefficients. Models were unadjusted (Model A) and adjusted for age, sex, hypertension, low-density-lipoprotein cholesterol (LDL-C), body mass index (BMI), smoking and alcohol consumption (Model B) providing estimates with 95% confidence intervals (CIs). For the association of spinal canal diameter with WMH volume, incidence rate ratios from zero-inflated negative binomial regression analysis were estimated. Modification effects of BMI and systolic blood pressure on the investigated associations were tested by including the product of both risk factors with each spinal canal width parameter additionally in the multivariable adjusted model, separately. Twelve participants with insufficient MRI image quality of the brain and five participants with insufficient MRI image quality of the spine were excluded. Brain volume measurements were missing in 36 participants due to insufficient brain segmentation resulting from suboptimal atlas registration. Spinal canal width measurements were missing in four participants at the C2/3 level due to insufficient coverage of the examined area by the field of view. Cobb angle measurements were missing in one participant due to incomplete image data (Fig. 1). Multiple imputation of missing data by predictions did not change the results substantially. All significant associations remained significant.

*P*-values <0.05 were considered to denote statistical significance. In addition, we evaluated the results according to a Bonferroni-adjusted significance level of *P* < 0.008 (0.05/6), due to multiple tests of two independent with three outcome variables.

## Ethics approval

This study was performed according to the Declaration of Helsinki. All study methods were approved by the ethics committee of the Bavarian Chamber of Physicians, Munich (S4: EC No. 99186 and for genetic epidemiological questions 05004, F4 and FF4: EC No. 06068). The MRI examination protocol was approved by the ethics committee of the Ludwig-Maximilian-University Hospital, Munich.

## Results

### Study population

The study sample consisted of 383 participants [57% male; age: 56.3 (±9.2) years]. The brain volume analysis sample for spinal canal width at the C2/3 level comprised 343 participants. The brain volume analysis sample for scoliosis comprised 346 participants. Mean total grey matter volume was 594 569 (±59 783) mm^3^ [intracranial volume (ICV)-corrected: 0.417 (±0.016)], mean total white matter volume was 586 323 (±59 430) mm^3^ [ICV-corrected: 0.411 (±0.016)], and mean brain ventricular volume was 243 731 (±42 280) mm^3^ [ICV-corrected: 0.171 (±0.028)]. Fifty-four (16%) participants had scoliosis. None of the participants had brain signal abnormalities other than WMH. None of the participants had myelopathic signal changes in the spinal cord. None of the participants had undergone cervical spine surgery. None of the participants had known dementia or anti-dementia medication. Further details and covariates such as blood pressure, blood lipid levels, BMI, smoking and alcohol consumption are presented in Table 1.

**Table 1:** Demographics table

	All *N* = 383	Without scoliosis *N* = 323^b^	With scoliosis *N* = 60^b^
**Age (years)**	56.3 (±9.2)	55.7 (±9.1)	59.1 (±8.9)
**Male sex**	218 (57%)	179 (57.2%)	36 (60%)
**Total grey matter volume (mm^3^)**	594 569 (±59 783)	595 456 (±61 268)	589 655 (±51 885)
**Total grey matter volume (ICV-corrected)**	0.417 (±0.016)	0.418 (±0.016)	0.412 (±0.015)
**Total white matter volume (mm^3^)**	586 323 (±59 430)	587 159 (±61 213)	581 725 (±49 583)
**Total white matter volume (ICV-corrected)**	0.411 (±0.016)	0.412 (±0.016)	0.406 (±0.016)
**Brain ventricular volume (mm^3^)**	243 731 (±42 280)	240 764 (±41 644)	260 396 (±42 368)
**Brain ventricular volume (ICV-corrected)**	0.171 (±0.028)	0.170 (±0.028)	0.182 (±0.027)
**Spinal canal width C2/3 (mm)**	11.7 (±1.2)	11.7 (±1.3)	11.7 (±1.2)
**BMI (kg/m^2^)**	28.0 (±4.7)	28.1 (±5.0)	27.7 (±3.5)
**Hypertension** ^a^	128 (33%)	100 (32%)	26 (43.3%)
**Systolic BP (mmHg)**	120.4 (±16.8)	120.0 (±17.4)	122.9 (±14.2)
**Diastolic BP (mmHg)**	75.3 (±10)	75.5 (±10.4)	74.1 (±8.0)
**Total cholesterol (mg/dl)**	218.3 (±35.9)	218 (±36.7)	219 (±32.8)
**HDL (mg/dl)**	62.3 (±17.6)	61.5 (±17.5)	66.4 (±18.6)
**LDL-C (mg/dl)**	139.8 (±32.4)	139.9 (±33.0)	139 (±29.6)
**Triglycerides (mg/dl)**	130.1 (±82.2)	133.2 (±85.6)	118.6 (±67.0)
**HbA1c (%)**	5.56 (±0.72)	5.55 (±0.73)	5.65 (±0.72)
**Fasting serum glucose (mg/dl)**	104 (±22.6)	103.9 (±23.4)	104.6 (±20)
**Glucose after 2- OGTT (mg/dl)**	113.3 (±40.9)	114.5 (±42.3)	105.2 (±32.7)
***Smoking status***			
**Never smoker**	140 (37%)	110 (35.1%)	26 (43.3%)
**Former smoker**	165 (43%)	137 (43.8%)	23 (38.3%)
**Current smoker**	78 (20%)	66 (21.1%)	11 (18.3%)
**Pack years**	12.6 (±17.8)	12.9 (±17.6)	12.4 (±20.1)
***Alcohol***			
**No consumption**	93 (24%)	78 (24.9%)	13 (21.7%)
**<20 g/day**	147 (38%)	118 (37.7%)	24 (40%)
**20–40 g/day**	75 (20%)	64 (20.5%)	8 (13.3%)
**>40 g/day**	68 (18%)	53 (16.9%)	15 (25%)

Data are means and standard deviations for continuous variables and counts and percentages for categorical variables.

OGTT, oral glucose tolerance test.

^a^Hypertension was defined as systolic blood pressure ≥ 140 mmHg, diastolic blood pressure ≥ 90 mmHg and/or use of anti-hypertensive medication.

^b^The brain volumes analysis sample for scoliosis comprised 292 participants without scoliosis and 54 with scoliosis.

### Associations of spinal canal diameter with brain volume

Spinal canal width measurements showed an excellent inter-reader reliability (ICC = 0.94) and excellent intra-reader reliability (ICC = 0.97).

Smaller spinal canal width at the C2/3 level was significantly associated with lower total grey matter volume [*β* = 0.003 (95% CI: 0.001; 0.004), *P* < 0.001. These effects were confirmed after adjustment for age, sex, hypertension, LDL-C, BMI, smoking and alcohol consumption [*β* = 0.001 (95% CI: 0; 0.002), *P* = 0.034]. However, after consideration of multiple testing with a Bonferroni-adjusted significance level of *P* < 0.008, this association did not stay significant after adjustment.

Smaller spinal canal width at the C2/3 level was significantly associated with lower total white matter volume [*β* = 0.003 (95% CI: 0.001; 0.004), *P* < 0.001]. These effects were confirmed after adjustment for age, sex, hypertension, LDL-C, BMI, smoking and alcohol consumption [*β* = 0.002 (95% CI: 0; 0.003), *P* = 0.012]. However, after consideration of multiple testing with a Bonferroni-adjusted significance level of *P* < 0.008, this association did not stay significant after adjustment.

**Figure 2:**
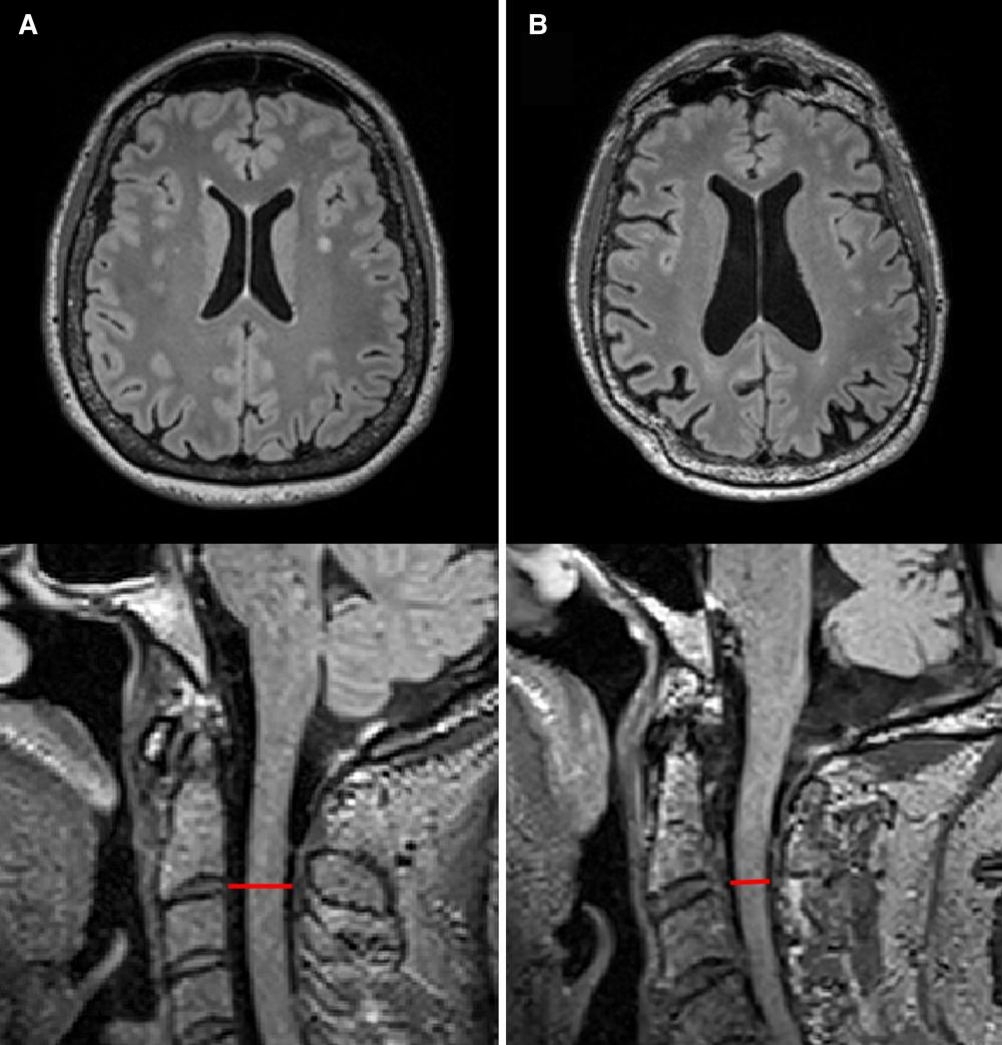
**MRI of the brain and cervical spine.** Axial T2-weighted FLAIR images of the brain (top row) and midsagittal T2-weighted FLAIR images of the spine at the cervical vertebrae (C) 2/3 level (bottom row). (**A**) A 51-year-old woman with a larger spinal canal diameter at the C2/3 level of 13.1 mm and non-dilated lateral ventricles. (**B**) A 69-year-old man with a smaller spinal canal diameter at the C2/3 level of 10.1 mm and larger lateral ventricles.

**Figure 3:**
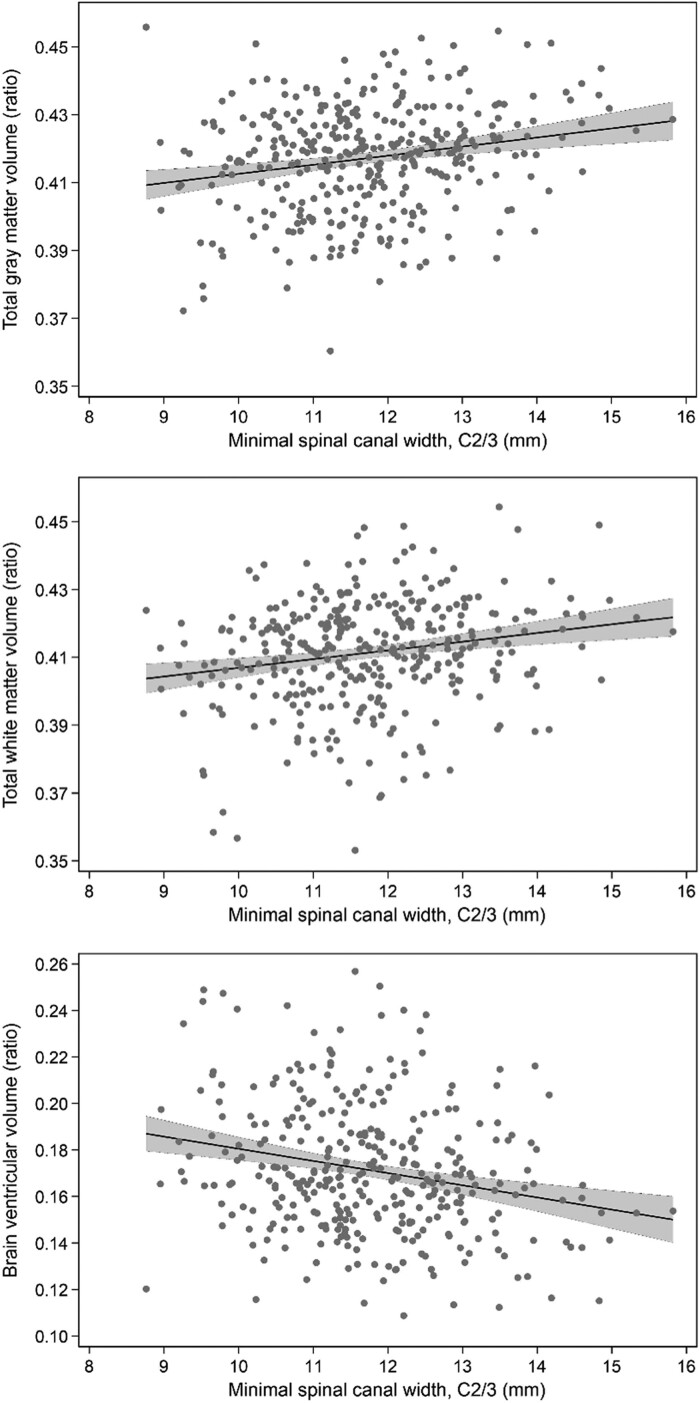
**Association of spinal canal width with brain volumes.** Visualization of predicted unadjusted grey matter volume (ratio), white matter volume (ratio) and brain ventricular volume (ratio) (*y*-axis) according to spinal canal width in mm at the cervical vertebrae (C) 2/3 level from a linear regression model with 95% CI (grey area) (Pearson correlation coefficients: *r* = 0.207, *P* < 0.001; *r* = 0.120, *P* < 0.001; *r* = −0.228, *P* < 0.001, respectively) (*N* = 343).

Smaller spinal canal width at the C2/3 level was significantly associated with higher brain ventricular volume [*β* = −0.005 (95% CI: −0.008; −0.003), *P* < 0.001] (Figs 2 and 3). These effects were confirmed after adjustment for age, sex, hypertension, LDL-C, BMI, smoking and alcohol consumption [*β* = −0.003 (95% CI: −0.005; −0.001), *P* = 0.006]. After consideration of multiple testing with a Bonferroni-adjusted significance level of *P* < 0.008, this association remained significant after adjustment. No association was modified by BMI or systolic blood pressure (all *P*-values >0.05).

Spinal canal width at the C2/3 level was not significantly associated with WMH volume before (*P* = 0.864) and after adjustment (*P* = 0.960) (Table 2).

**Table 2:** Association of spinal canal width with total grey matter volume, total white matter volume, brain ventricular volume and WMH volume

		Spinal canal width C2/3	
		Estimate (95% CI)	*P*-value
**Total grey matter volume**	**A**	0.003 (0.001; 0.004)	<0.001
	**B**	0.001 (0; 0.002)	0.034
**Total white matter volume**	**A**	0.003 (0.001; 0.004)	<0.001
	**B**	0.002 (0; 0.003)	0.012
**Brain ventricular volume**	**A**	−0.005 (−0.008; −0.003)	<0.001
	**B**	−0.003 (−0.005; −0.001)	0.006
WMH volume*	**A**	0.80 (0.06; 10.52)	0.864
	**B**	0.93 (0.06; 14.1)	0.960

Estimates are β-coefficients from linear regression or *incidence rate ratios from zero-inflated negative binomial regression models. A: Unadjusted. B: Adjusted for age, sex, hypertension, LDL-C, BMI, smoking and alcohol consumption.

Spinal canal width at the L1-S1 levels was not significantly associated with white matter volume, grey matter volume, brain ventricular volume or WMH volume (Supplementary Table 1).

### Associations of scoliosis with brain volume

Cobb angle measurements showed an excellent inter-reader reliability (ICC = 0.93) and excellent intra-reader reliability (ICC = 0.98).

**Figure 4:**
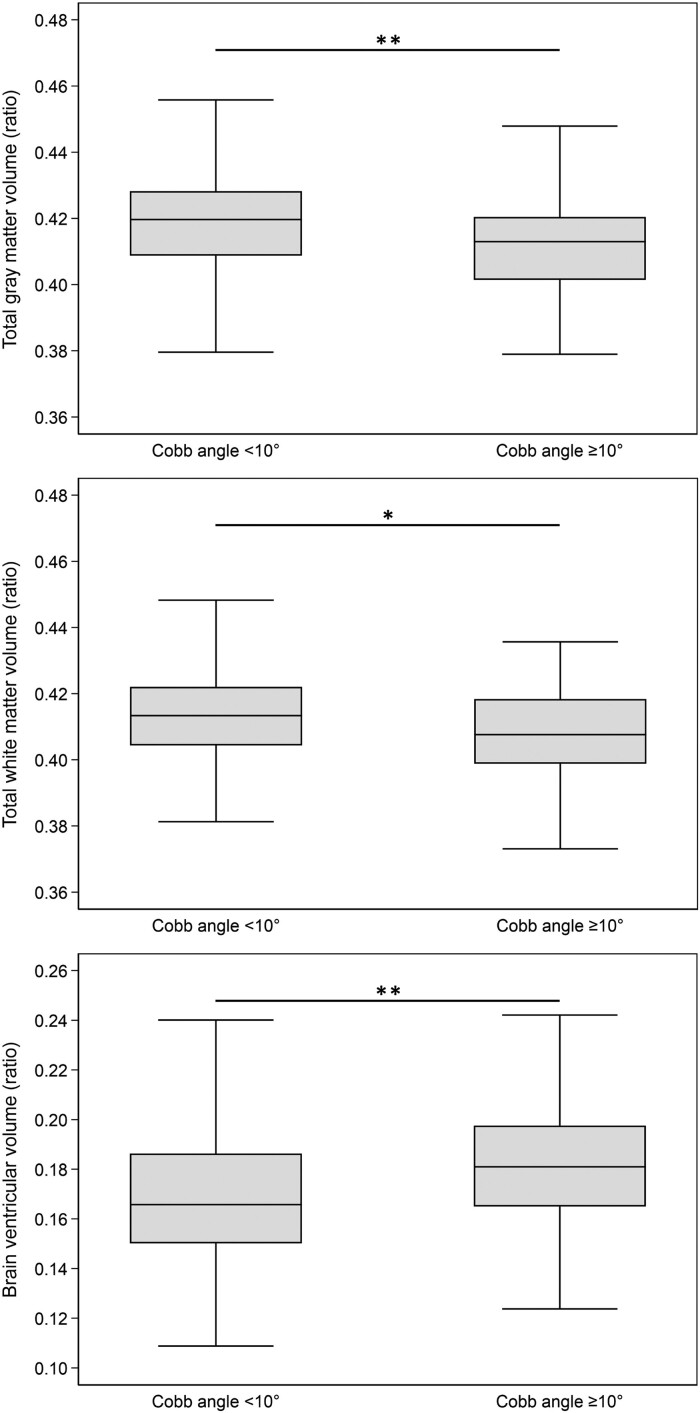
**Comparisons between participants without and with scoliosis.** Unadjusted comparisons (*t*-tests) of grey matter volume (ratio), white matter volume (ratio) and brain ventricular volume (ratio) (*y*-axis) between participants without scoliosis (Cobb angle of <10°, *N* = 292) and with scoliosis (Cobb angle of ≥10°, *N* = 54) (*x*-axis). Participants with a Cobb angle of ≥10° had significantly smaller grey matter volumes (**P = 0.005), smaller white matter volumes (*P = 0.011) and larger brain ventricular volumes (**P = 0.003).

Participants with scoliosis (Cobb angle of ≥10°) had significantly lower total grey matter volumes [*β* = −0.006 (95% CI: −0.011; −0.002), *P* = 0.005], significantly lower total white matter volumes [*β* = −0.006 (95% CI: −0.011; −0.001), *P* = 0.011] and significantly larger brain ventricular volumes [*β* = 0.012 (95% CI: 0.004; 0.02), *P* = 0.003] than participants without scoliosis (Cobb angle of <10°) (Fig. 4). However, these associations were attenuated after adjustment for age, sex, hypertension, LDL-C, BMI, smoking and alcohol consumption for total grey matter volumes [*β* = −0.003 (95% CI: −0.007; 0.001), *P* = 0.137], total white matter volumes [*β* = 0.004 (95% CI: −0.008; 0.001), *P* = 0.099] and brain ventricular volumes [*β* = 0.006 (95% CI: 0; 0.013), *P* = 0.065] (Table 3).

**Table 3:** Association of scoliosis (Cobb angle of ≥10°) with total grey matter volume, total white matter volume and brain ventricular volume

		Participants with scoliosis (Cobb angle of ≥10°) versus participants without scoliosis (Cobb angle of <10°)	
		Estimate (95% CI)	*P*-value
**Total grey matter volume**	**A**	−0.006 (−0.011; −0.002)	0.005
	**B**	−0.003 (−0.007; 0.001)	0.137
**Total white matter volume**	**A**	−0.006 (−0.011; −0.001)	0.011
	**B**	−0.004 (−0.008; 0.001)	0.099
**Brain ventricular volume**	**A**	0.012 (0.004; 0.02)	0.003
	**B**	0.006 (0; 0.013)	0.065

Estimates are β-coefficients from linear regression models. A: Unadjusted. B: Adjusted for age, sex, hypertension, LDL-C, BMI, smoking and alcohol consumption.

Participants with scoliosis (Cobb angle of ≥10°) did not have significantly higher WMH volumes than participants without scoliosis (Cobb angle of <10°) before (*P* = 0.999) and after adjustment (*P* = 0.976).

After adjustment, higher Cobb angle values were associated with lower grey matter volume [*β* = −0.0002 (95% CI: −0.0005; 0), *P* = 0.049], lower white matter volume [*β* = −0.0003 (95% CI: −0.0006; 0), *P* = 0.030] and higher brain ventricular volume [*β* = 0.0005 (95% CI: 0.0001; 0.001), *P* = 0.015] (Table 4). However, after consideration of multiple testing with a Bonferroni-adjusted significance level of *P* < 0.008, these associations did not stay significant after adjustment. Cobb angle values were not significantly associated with WMH volumes before (*P* = 0.987) and after adjustment (*P* = 0.962).

**Table 4:** Association of lateral spinal deformity (Cobb angle) with total grey matter volume, total white matter volume, brain ventricular volume and WMH volume

		Lateral spinal deformity (Cobb angle)	
		Estimate (95% CI)	*P*-value
**Total grey matter volume**	**A**	−0.0004 (−0.0007; −0.0001)	0.005
	**B**	−0.0002 (−0.0005; 0)	0.049
**Total white matter volume**	**A**	−0.0004 (−0.0007; −0.0001)	0.005
	**B**	−0.0003 (−0.0006; 0)	0.030
**Brain ventricular volume**	**A**	0.0008 (0.0003; 0.0013)	0.002
	**B**	0.0005 (0.0001; 0.001)	0.015
WMH volume*	**A**	1 (0.57; 1.74)	0.987
	**B**	0.99 (0.54; 1.79)	0.962

Estimates are β-coefficients from linear regression or *incidence rate ratios from zero-inflated negative binomial regression models. A: unadjusted. B: adjusted for age, gender, hypertension, LDL-C, BMI, smoking and alcohol consumption.

In a subgroup analysis of participants with scoliosis (*N* = 60), higher Cobb angle values were associated with lower grey matter volume [*β* = −0.0002 (95% CI: −0.0008; 0.0003), *P* = 0.419], lower white matter volume [*β* = −0.0003 (95% CI: −0.0009; 0.0003), *P* = 0.267] and higher brain ventricular volume [*β* = 0.0005 (95% CI: −0.0004; 0.0015), *P* = 0.259]. However, these associations were not significant.

Linear regression model revealed no adjusted mean difference of spinal canal width at the C2/3 level between participants without and with scoliosis [*β* = 0.05 mm; 95% CI −0.30; 0.40, *P* = 0.779].

## Discussion

In this population-based study, we evaluated the association of spinal MR imaging morphology with brain volume. Confounder adjusted analysis showed that smaller spinal canal width at the C2/3 level was significantly associated with lower grey matter, lower white matter and higher ventricular volumes. However, after consideration of multiple testing, only the association of spinal canal width at the C2/3 level with ventricular volumes remained significant after adjustment. Cobb angle values showed a significant association with grey matter, white matter and ventricular volumes after adjustment. Brain matter volumes were significantly decreased, and brain ventricular volumes were significantly increased in participants with scoliosis compared with participants without scoliosis. However, these effects were attenuated after adjustment. Spinal canal width at the C2/3 level and scoliosis were not associated with WMH volume before and after adjustment.

Our findings support the notion that the compliance of the spinal CSF compartment may be influenced by spinal morphology. In a pilot study of 10 participants with idiopathic normal pressure hydrocephalus and 32 healthy controls, maximal systolic craniospinal CSF flow rates were significantly reduced (*P* < 0.01) and spinal canal diameters at the C2/3 level significantly decreased (*P*< 0.001) in patients with idiopathic normal pressure hydrocephalus. Additionally, a significantly positive correlation between maximal CSF flow rates and spinal canal width at the C2/3 level was found, showing a lower maximal CSF flow with lower spinal canal width at the C2/3 level (*R* = 0.47; *P* < 0.05).^15^ An animal model demonstrated significant ventricular enlargement (*P*< 0.002) in cats with sub-chronic cervical stenosis at the C2 level compared with healthy controls.^13^ A longitudinal study of 15 patients with spinal cord injury (*n* = 12 cervical and/or thoracic level) and 18 healthy controls showed a significant acceleration of ventricular enlargement (third ventricle: *P* = 0.017; fourth ventricle: *P* = 0.006) compared with healthy controls.^28,29^

A further potential mechanism causing brain matter loss might constitute retrograde demyelination of the brain induced by spinal canal compression at the C2/3 level. In 27 patients with cervical spinal canal stenosis and 24 healthy controls, it was shown that cervical spinal canal stenosis caused by spondylosis may lead to brain atrophy through retrograde demyelination affecting the white and grey matter of the sensorimotor cortex.^30^

In addition to mechanical components, disturbances of the glymphatic system caused by impaired drainage into the spinal subpial compartment due to reduced spinal canal width at the C2/3 level might potentially contribute to brain matter loss. The glymphatic system is composed of a network of perivascular channels made of astroglia cells, which among others play a key role in brain fluid clearance, transport of nutrients and waste removal during sleep.^9-11^ Disturbances of the glymphatic system have been associated with brain matter loss and ventricular enlargement in idiopathic normal pressure hydrocephalus and Alzheimer’s disease.^12^ The hypothesis that reduced spinal canal width could potentially promote the development of WMH was not confirmed in the present study.

The present study adds to the field by providing a comprehensive, confounder-adjusted evaluation of the association between spinal canal width at the C2/3 level and brain volumes in a population-based sample of 383 participants. Our results strengthen the notion that smaller spinal canal width at the C2/3 level might be associated with brain ventricular dilation and brain matter loss. Additionally, it provides new insights in the potential effect of scoliosis on brain ventricular enlargement and brain atrophy. Our results indicate that scoliosis may have a negative impact on the compliance of the spinal CSF space. Scoliosis may impair the outflow of systolic CSF volume into the spinal canal, potentially resulting in ventricular enlargement and brain matter loss. To the best of our knowledge, this potential association has not been previously addressed.

The results of this study need to be interpreted in the light of its limitations. This study is based on structural measurements of the brain and spine only, with spinal canal diameter as a readily available and easy-to-measure parameter. Further studies assessing morphological measurements such as the cross-sectional area, Pavlov’s ratio or myelon-corrected relative spinal canal width, as well as additional functional parameters such as CSF flow rates, are warranted.^31^ After consideration of multiple testing with a Bonferroni-adjusted significance level of *P* < 0.008, associations of spinal canal width at the C2/3 level with grey matter and white matter volumes did not stay significant after adjustment for age, sex, hypertension, LDL-C, BMI, smoking and alcohol consumption, as well as associations of scoliosis with brain volumes. None of the participants had known dementia or anti-dementia medication; however, a thorough assessment of symptoms of normal pressure hydrocephalus was not performed in this study. Due to time restrictions of the whole-body MRI protocol of the KORA MRI study, T1-weighted sequences of the brain were not acquired. Selected cortical regions, especially the central region, and total white matter volume tend to be underestimated by the warp-based automated brain volumetric approach based on T2-weighted FLAIR images used in this study. Scoliosis measurements were acquired on MRI data sets with participants in supine position. However, it was shown that measurements of the spine in standing position and supine position are comparable when participants are positioned correctly.^32,33^ All participants in this study were positioned for MRI according to a standardized protocol.

## Conclusion

In conclusion, smaller spinal canal width at the level of C2/3 was associated with lower brain matter and higher ventricular volumes in this population-based study. Participants with scoliosis had lower brain matter and higher ventricular volume. However, these effects were attenuated after adjustment. These characteristics of spinal morphology might constitute independent risk factors for neurodegenerative disorders such as idiopathic normal pressure hydrocephalus and should be further evaluated in future studies.

## Supplementary Material

fcae365_Supplementary_Data

## Data Availability

The data underlying this article will be shared on reasonable request to the corresponding author.
