# Supplementary material for: The spine-brain axis: is spinal anatomy associated with brain volume?
Source: Brain Commun. 2024 Oct 22;6(5):fcae365. doi: 10.1093/braincomms/fcae365 (PMC11503949; doi:10.1093/braincomms/fcae365)
Supplement: fcae365_Supplementary_Data [file fcae365_supplementary_data.docx]

**The spine-brain axis – is spinal anatomy associated with brain volume?**

**Supplemental Material**

**Supplementary Table 1**

|  | | Minimal spinal canal width (lumbosacral) | | | | | | | | | |
| --- | --- | --- | --- | --- | --- | --- | --- | --- | --- | --- | --- |
|  |  | **L1/2** | | **L2/3** | | **L3/4** | | **L4/5** | | **L5/S1** | |
|  |  | **β (95% CI)** | **p-value** | **β (95% CI)** | **p-value** | **β (95% CI)** | **p-value** | **β (95% CI)** | **p-value** | **β (95% CI)** | **p-value** |
| **Total gray matter volume** | **A** | 0 (-0.001; 0.001) | 0.446 | 0 (-0.001; 0.001) | 0.484 | 0 (-0.001; 0.001) | 0.943 | 0 (-0.001; 0.001) | 0.826 | 0 (-0.001; 0) | 0.213 |
|  | **B** | 0 (0; 0.001) | 0.356 | 0 (0; 0.001) | 0.312 | 0 (0; 0.001) | 0.225 | 0 (0; 0.001) | 0.143 | 0 (0; 0.001) | 0.29 |
| **Total white matter volume** | **A** | 0 (-0.001; 0.001) | 0.965 | 0 (-0.001; 0.001) | 0.987 | 0 (0; 0.001) | 0.544 | 0 (0; 0.001) | 0.375 | 0 (-0.001; 0.001) | 0.785 |
|  | **B** | 0.001 (0; 0.001) | 0.194 | 0.001 (0; 0.001) | 0.200 | 0.001 (0; 0.001) | 0.119 | 0.001 (0; 0.001) | 0.077 | 0.001 (0; 0.001) | 0.022 |
| **Brain ventricular volume** | **A** | 0 (-0.001; 0.002) | 0.685 | 0 (-0.001; 0.002) | 0.687 | 0 (-0.001; 0.001) | 0.762 | 0 (-0.001; 0.001) | 0.532 | 0 (-0.001; 0.001) | 0.584 |
|  | **B** | -0.001 (-0.002; 0) | 0.190 | -0.001 (-0.002; 0) | 0.176 | -0.001 (-0.002; 0) | 0.102 | -0.001 (-0.002; 0) | 0.057 | -0.001 (-0.002; 0) | 0.045 |
| **WMH volume*** | **A** | 1.11 (0.2; 6.25) | 0.904 | 1.13 (0.25; 5.22) | 0.872 | 1.06 (0.3; 3.74) | 0.931 | 1.08 (0.37; 3.18) | 0.888 | 1.13 (0.37; 3.41) | 0.834 |
|  | **B** | 1.00 (0.17; 5.91) | 1.000 | 1.10 (0.22; 5.53) | 0.910 | 1.04 (0.27; 3.93) | 0.957 | 1.07 (0.35; 3.28) | 0.907 | 1.09 (0.33; 3.66) | 0.884 |

**Supplementary Table 1:** Association of spinal canal width with total gray matter volume, total white matter volume, and brain ventricular volume. Estimates are β-coefficients from linear regression models or *incidence rate ratios (IRR) from zero-inflated negative binomial regression models. **A:** Unadjusted. **B:** Adjusted for age, sex, hypertension, LDL-C, BMI, smoking, alcohol consumption.

**Supplementary Figure 1**


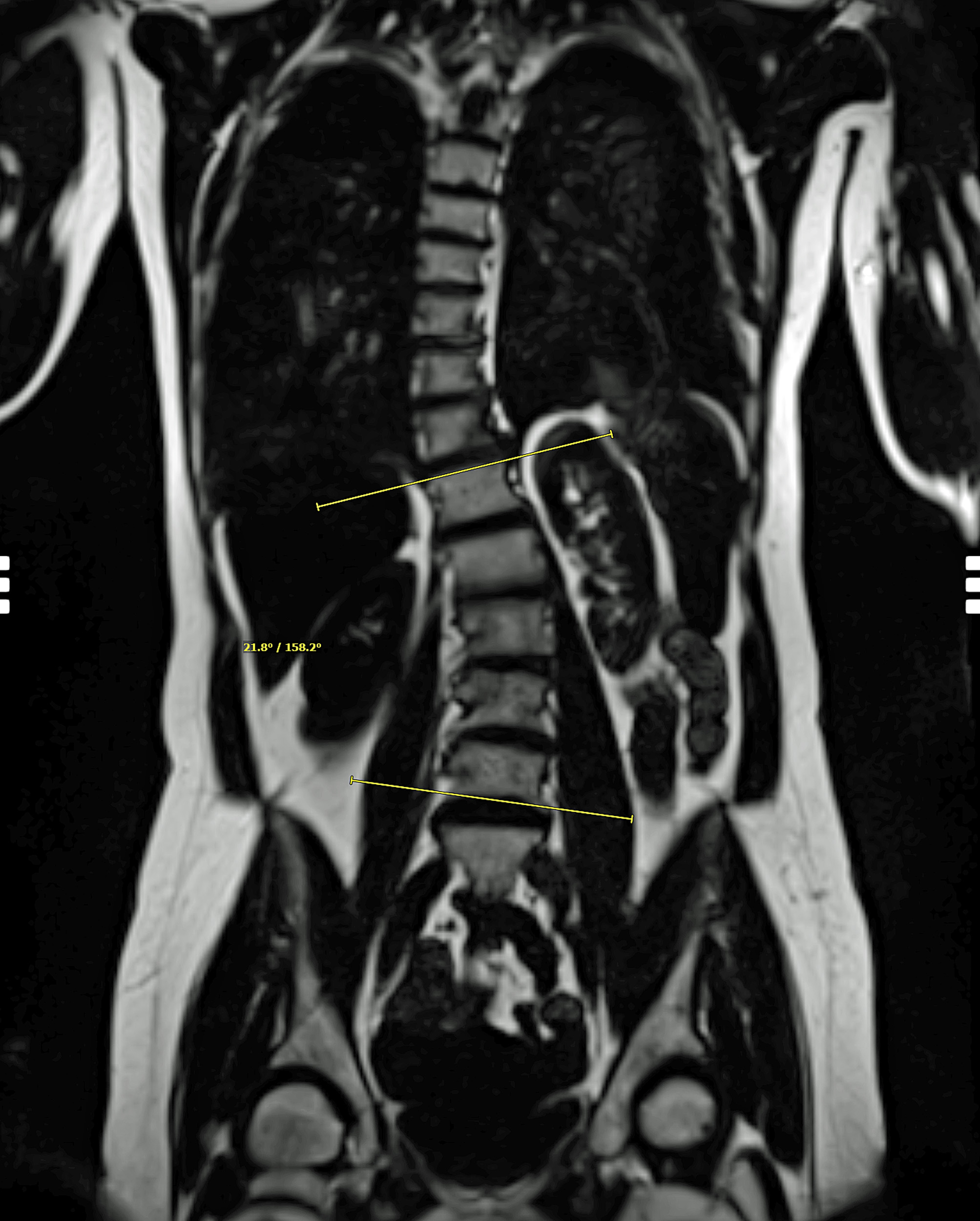


**Supplementary Figure 1:** Coronal T1-weighted VIBE DIXON image of the spine. To evaluate scoliosis the Cobb angle was measured, which was created by an extension line from the upper endplate of the uppermost vertebral body and lower endplate of the lowermost vertebral body involved in lateral spinal deviation.
